# Erratum, Vol. 9, September 27 Release

**DOI:** 10.5888/pcd10.120065e

**Published:** 2013-05-23

In the article “Factors Associated With Poor Glycemic Control or Wide Glycemic Variability Among Diabetes Patients in Hawaii, 2006-2009,” one of the funding sources for the study was not mentioned in the Acknowledgments section. The additional funding source is ARRA grant no. 3P20MD000173–09S1 from the National Institute on Minority Health and Health Disparities. The correction was made to our website on May 15, 2013, and appears online at http://www.cdc.gov/pcd/issues/2012/12_0065.htm. We regret any confusion or inconvenience this error may have caused.

